# The Combined Effects of Tai Chi, Resistance Training, and Diet on Physical Function and Body Composition in Obese Older Women

**DOI:** 10.1155/2014/657851

**Published:** 2014-12-28

**Authors:** S. A. Maris, D. Quintanilla, A. Taetzsch, A. Picard, J. Letendre, L. Mahler, I. Lofgren, F. Xu, M. J. Delmonico

**Affiliations:** ^1^Department of Kinesiology, University of Rhode Island, Kingston, RI 02881, USA; ^2^Department of Nutrition and Food Sciences, University of Rhode Island, Kingston, RI 02881, USA; ^3^Department of Communicative Disorders, University of Rhode Island, Kingston, RI 02881, USA

## Abstract

Obesity is a major health problem in the USA, especially in minority populations over the age of 60 years, and the aging process can cause adverse effects on physical function. Previous research has shown that Tai Chi, resistance training (RT), and diet result in overall health improvements. However, the combination of these specific interventions has yet to be translated to obese older women in an urban setting. The purpose of this study was to examine a combined intervention on the primary outcomes of physical function and body composition. Using a nonrandomized design, 26 obese women (65.2 ± 8.1 years) completed a 12-week intervention; participants were assigned to an intervention (EXD) group or a control (CON) group. The EXD group (*n* = 17) participated in Tai Chi, RT, and a dietary session. The CON group (*n* = 9) was asked to continue their normal lifestyle. Timed up and go (TUG) time was reduced by 0.64 ± 2.1 seconds (*P* = 0.04) in the EXD group while the CON group saw a borderline significant increase of 0.71 sec (*P* = 0.051). The combined intervention helped improve performance on TUG time, but there were no significant increases in other body composition or function measures.

## 1. Introduction

The prevalence of obesity, defined as a body mass index (BMI) greater than 30 kg/m^2^, has doubled in the past 10 years in the general population and estimates indicate that 35% of adults over the age of 65 are obese in the USA [1]. Obese individuals are at a greater risk of developing health conditions including hypertension, cardiovascular disease, diabetes and some forms of cancer [2, 3]. Previous research [4] has shown that women have a higher prevalence of obesity compared to men, and the rates of obesity greatly differ across different racial groups. Over the age of 60 years, the prevalence rates of obesity for non-Hispanic White women, non-Hispanic Black women, Hispanic women, and non-Hispanic Asian women are 32.8%, 56.6%, 44.4%, and 11.4%, respectively [5].

In older women, obesity accelerates the decline of physical function that is associated with aging and has adverse effects on chronic disease risk [6]. The aging process has a negative effect on physical function and results in decreased muscular strength, and this decline in muscular strength associated with aging is known as sarcopenia [7, 8]. Sarcopenia is the age-related loss of muscle mass, and the decrease in muscular strength is considered to be a contributor to the decline in overall physical functioning in the older adult population [8]. Effective approaches targeting active lifestyles are needed as older adults have four times the physical limitations when compared to individuals less than 60 years of age [9].

Resistance training (RT) is a frequently used exercise modality in obese individuals and has been shown to improve muscle mass and strength, physical function, and body composition in older women [10–14]. However, RT is not considered an aerobic activity; in contrast, Tai Chi exercise is comparable to aerobic activities [15, 16]. Aerobic activities have greater influences on obesity reduction and promote greater weight loss when compared to anaerobic activities such as RT. Previous studies utilizing Tai Chi as an exercise intervention have demonstrated significant improvements in physical function and facilitate weight loss [17–21]. Resistance training and Tai Chi have also been found to have a very high adherence rate in older adults due to the low risk of injury associated when performed at a moderate intensity [12, 13, 20–22]. Along with increasing physical activity, older adults should improve their diet to augment the effects of exercise [2].

Combining an exercise program with a behavioral-based dietary intervention has been shown to significantly improve function and body composition [6, 23–25]. Previous dietary research has shown that the Dietary Approach to Stop Hypertension (DASH) diet improves total diet quality, blood pressure, and can facilitate weight loss [23, 26, 27]. The DASH diet has been shown to be an effective and reliable intervention tool in obese older adults [23, 25, 28]. Furthermore, the modified DASH diet (<35% fat versus <27% fat) combined with exercise results in significant decreases in fat mass and results in significant weight loss in older adults [15, 25].

There have been numerous studies that show combining exercise forms with a dietary intervention results in improved physical function, muscle strength, and body composition [6, 15, 24, 25, 29]. However, there is a lack of translational research on Tai Chi and RT in combination with dietary changes in an urban setting. In addition, a study combining these individually demonstrated interventions has not been evaluated in obese older women in an urban setting. We hypothesized that physical function, muscular strength, and body composition would significantly improve in the intervention (EXD) group as a result of a 12-week intervention compared to a control (CON) group. Thus, the purpose of this study was to examine the effects of Tai Chi, diet, and RT in obese older women on measures of physical and muscle function and body composition.

## 2. Methods

### 2.1. Study Design

This study used a nonrandomized intervention design to study changes in physical function and body composition in 26 obese older women. The intervention consisted of a 12-week design with a control group used as a comparison. The study took place at an urban senior center in Providence, Rhode Island. This study was approved by the Institutional Review Board at the University of Rhode Island, and a written informed consent was obtained from each participant prior to the start of the study.

### 2.2. Participants

Women living in the surrounding communities (within 1 mile of the senior center) were recruited for this study via flyers, press releases to local newspapers, and “word-of-mouth” at the local senior center. Eligibility criteria included (1) women ages of 50–80 years, (2) BMI of 30.0 to 50.0 kg/m², (3) being not involved in an exercise program in the past 6 months, and (4) postmenopausal women. The exclusionary criteria included (1) failure to provide informed consent, (2) significant or suspected cognitive impairment, (3) severe hearing loss, speech disorder, language barrier, or visual impairment, (4) progressive, degenerative neurologic disease, (5) terminal illness with life expectancy of <12 months as determined by physician, (6) severe pulmonary disease, uncontrolled diabetes, blood pressure, or anemia, (7) being not medically stable, (8) major surgery within 6 months, (9) significant cardiovascular disease, and (10) inability to safely engage in exercise. All of the inclusion and exclusion criteria were obtained via self-report, but height, weight, and BMI were measured during an orientation session. Verification of other exclusion criteria was accomplished by requesting medical clearance from each participant's primary care physician if the participant had one. However, medical clearance was not a requirement for the study as some participants were of lower income and did not have a primary care physician.

### 2.3. Outcome Measures

All testing procedures were performed at the senior center (intervention site) under standardized conditions to reduce variability in the results. Participants were given a familiarization trial before all physical performance testing procedures to ensure understanding of the test and to allow for practice.

#### 2.3.1. Physical Function

The primary measure of physical function was the timed up and go (TUG) test. The TUG test has been shown to be a valid predictor of falls and mobility of the older adult population [30]. The TUG test requires that the participants rise from a chair and walk at normal gait speed around a cone eight feet away and then return and sit back down.

Another measure of function was the short physical performance battery (SPPB) that includes a standing balance test, 4-meter usual gait speed, and timed 5-chair stands. Each test was scored and analyzed individually with a score of 0–4, and a total score out of 12 was calculated. The SPPB is a portable and reliable measure that has been shown to be a strong predictor of mortality and overall function [31].

#### 2.3.2. Flexibility

The chair-sit-and-reach test is a measure of flexibility used frequently in older adults [32]. This test required the participant to sit on the edge of a chair with one knee bent and the other knee extended straight in front, while reaching towards the toes. The score was the number of centimeters short of reaching the toes (negative number) or beyond the toes (positive number).

#### 2.3.3. Muscle Function and Strength

Grip strength is a valid and reliable method and has been shown to be successful in measuring upper body strength in older adults [33]. Grip strength was measured using a hand-grip dynamometer (Jaymar Dynamometer, J.A. Preston, Corp., Jackson, MS) in the participants nondominant hand. The hand dynamometer is considered to be a very reliable tool and can be performed with little or no risk for the participants [34].

Knee extensor isometric strength was determined by using a handheld manual muscle dynamometer (Nicholas Manual Muscle Tester—Lafayette Instrument Company, Lafayette, IN). This was measured as the peak amount of force that the examiner had to exert to break the isometric contraction held by the participant [35]. The device has been successful in determining strength in the older adult population and it is a portable, easy to use device [28]. The value recorded was leg extensor torque (kg-m) in order to account for variations in leg length.

#### 2.3.4. Anthropometrics

Height and weight were measured with a stadiometer (Webb City, MO, USA) to calculate BMI and were following a 12-hour fast. Waist and hip measures were determined by utilizing a standard tape measure with an attached tensometer. Body composition was measured using a simple foot-to-foot bioelectrical impedance device (Tanita BF-556). This test has been shown to be a valid and reliable measure of body composition while having few associated risks [36].

#### 2.3.5. Other Measures

The participants were asked to complete surveys about physical activity levels and dietary quality. The Yale Physical Activity Survey was used to estimate energy expenditure per week and time spent performing physical activity [37]. Dietary quality and patterns were assessed using the Dietary Screening Tool (DST). The DST was created to assess nutrition status and classify participants in relation to nutrition risk among older adults [38].

### 2.4. Group Assignment

Since this project was a translational study designed to benefit the participants, a nonrandomized design was chosen. After baseline testing was completed, participants were placed into either the EXD group or the CON group by the order in which they finished testing. Due to the limited space at the local senior center, there was a limit on how many participants could be placed in the EXD group. Those participants who wished to be involved in the study after the EXD group was full were asked to be in the CON group. The EXD group received all three aspects of the intervention, while the CON group was asked to maintain their normal lifestyle.

### 2.5. Intervention

#### 2.5.1. DASH Dietary Education Intervention

A 45-minute behavior-based dietary education session was held once a week for the 12-week intervention period. The dietary sessions were instructed and led by a registered dietitian. A modified DASH-based diet was used as the diet plan and has been shown to have a high compliance rate with older adults [23, 26]. Dietary logs were used to evaluate participants' compliance throughout the 12 weeks and to provide feedback to the participants.

#### 2.5.2. Tai Chi Exercise Intervention

Participants in the EXD group participated in Tai Chi exercise sessions 3 times per week for 12 weeks. The Tai Chi exercise was led by trained study staff who were trained in Tai Chi by an experienced principal investigator before the start of the intervention. The modified 24-movement Yang style form of Tai Chi was used since it has been shown to be an effective form of Tai Chi through previous research [15]. The Tai Chi sessions lasted approximately 45 minutes and included 10-minute warm up and 35 minutes of practice and exercise and were followed by a 5-minute cooldown period.

#### 2.5.3. RT Exercise Intervention

The RT program was based on the American College of Sports Medicine RT Guidelines [3], and each session included approximately 45 minutes. The RT sessions were led by trained study staff who were trained by a principal investigator in the protocol prior to the beginning of the study. The program incorporated elastic tubing which may help avoid musculoskeletal injuries and can be used as a practical method of achieving strength gains in older adults [14]. Seven total upper and lower body exercises for major muscles were done with 1–3 sets of 10–15 repetitions for each exercise. The goal was for the exercise intensity to be moderate, with the final repetitions being at or near maximum voluntary effort. This intensity level has been shown to have positive effects on physical functioning in older adults [28].

### 2.6. Statistical Analysis

To assess our main hypothesis, the changes from baseline descriptive measures were analyzed to define differences between the groups. Independent samples *t*-tests for continuous data and a Fisher's exact test were used for categorical data. Next, the Shapiro Wilk test was used to determine if the data for changes in the outcome variables were normally distributed. If the data were normally distributed, a paired *t*-test was run to examine within-group changes from baseline. If the data were not normally distributed (TUG test and SPPB) a nonparametric equivalent was used, the signed rank test. A univariate analysis with outliers defined as data point 3 deviations higher or lower than the mean and Cook's distance [39] test (>1.0 for as a cut-point) were conducted with the data to determine outliers, but no outliers for changes in the main outcome variables were found. Analyses of covariance tests were utilized to examine the pre-/postchanges between the two groups, adjusted for baseline values. All data were expressed as means (±standard deviation).

## 3. Results

**Figure 1:**
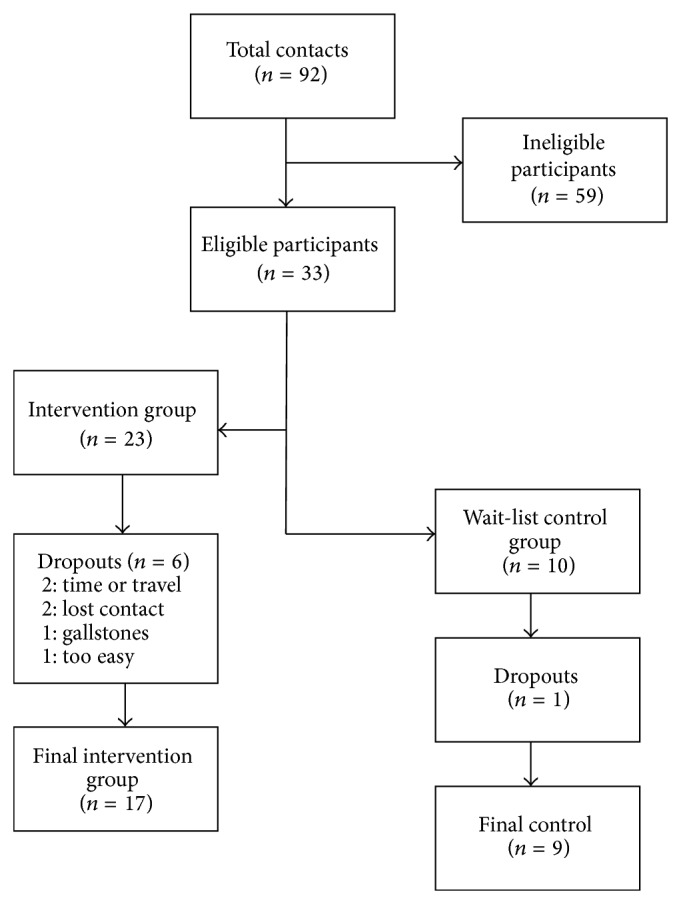
Flow chart of participants throughout the study. The total analytical sample consisted of 26 participants, with 17 in the EXD group and 9 in the control group.

Figure 1 depicts the flow of participants that took place throughout the study. The total analytic sample consisted of 26 participants, with 17 in the EXD group, BMI = 38.8 (5.1) kg/m^2^, and 9 in the CON group, BMI = 36.6 (3.4) kg/m^2^. The mean attendance for all aspects of the intervention sessions was 67.5% in the EXD group.

Table 1 describes the baseline characteristics of the two groups. There were significant differences between groups in time spent performing physical activity [7965.5 (5696.5) kcal/week in the EXD group versus 3100.4 (3657.5) kcal/week in the CON group; *P* = 0.038] and average waist circumference [115.0 (8.9) cm in the EXD group versus 106.8 (9.8) cm in the CON group; *P* = 0.042].

**Figure 2:**
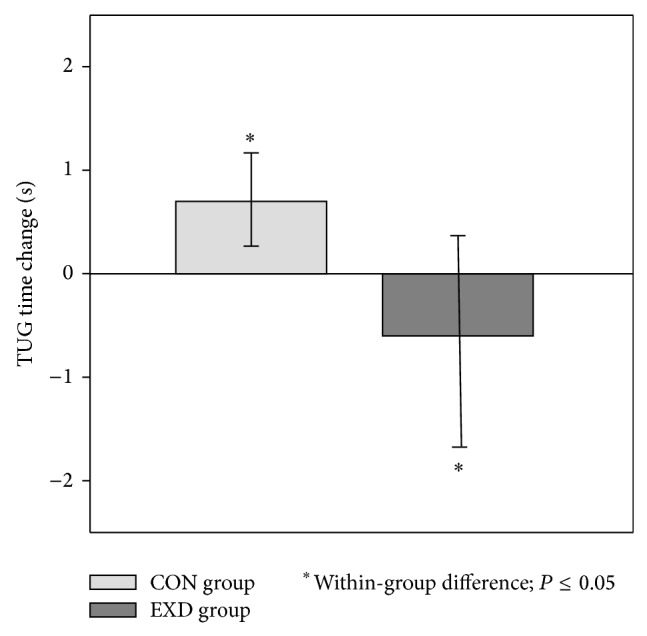
Changes in the EXD and CON groups in TUG time change with the 12-week intervention. The EXD group improved TUG time by of 0.6 (2.1) sec with the 12-week intervention (*P* = 0.04) and the CON group saw a decline of 0.7 (SD = 0.9) after the 12 weeks (*P* = 0.05). ^*^Significant changes within groups (*P* < 0.05). TUG: timed up and go. All data were analyzed using analysis of covariance adjusted for baseline values. All data are expressed as least squared means (standard error).

Figure 2 depicts the changes that occurred in TUG time in both groups with the 12-week intervention. There were significant within-group changes in TUG time in the EXD and the CON groups, as the EXD group saw an improvement of −0.6 (2.1) sec (*P* = 0.04), and the CON group saw a decline of 0.7 (0.9) seconds with the 12-week period (*P* = 0.05).

Table 2 shows the changes that occurred from baseline to 12 weeks in both groups regarding function and muscle strength. There were no significant changes that occurred after baseline regarding between-group differences for muscle strength or SPPB scores. However, the EXD group had the tendency to improve flexibility by 2.3 (5.4) cm with the 12-week intervention (*P* = 0.07), while the CON group did not significantly change (*P* = 0.51).

Table 3 reports the changes that occurred in body composition between both groups. The EXD group had an unexpected increase in waist to hip ratio by 0.03 (0.01) (*P* = 0.04), but the CON group did not significantly change (*P* > 0.05). There were no other significant changes in measures of body composition.

## 4. Discussion

The major finding of the study was that significant improvements were observed in the TUG, a measure of mobility, after a 12-week Tai Chi, RT, and diet intervention in a mostly minority population. There was also a tendency for improved flexibility as assessed by a sit-and-reach test. To our knowledge, the current translational study is the first to test the combined interventions of Tai Chi, RT, and diet on physical function and body composition in obese older women living in an urban community setting.

The major finding of this current study has shown that TUG performance improved in the EXD group and declined in the CON group. The reduction in TUG time of 0.6 seconds in the EXD group was a 5.7% improvement from baseline in the EXD group, while an increase of 0.7 seconds was a worsening of 8.7% in the CON group. These findings in TUG improvement confirm the results of previous studies utilizing Tai Chi [18]. Tai Chi alone and Tai Chi combined with other interventions have been shown to result in positive improvements in function [15, 18, 22]. These changes in TUG time could be attributed to the tendency to improve flexibility, as well as improvements in balance that were seen in previous studies incorporating Tai Chi [23]. It is important to monitor and improve physical function as it is a measure of functional independence in the elderly, and improving measures of function can assist with completion of everyday tasks [40]. By maintaining function and fitness capacity, older adults can continue to live independently and can better accomplish everyday activities [40]. Maintaining independence and a healthy lifestyle can reduce economic and personal burdens that are commonly associated with aging [40].

Unexpectedly, muscle strength did not significantly improve in the EXD group. The lack of change in muscle strength was an unexpected finding because previous studies observed improvements in muscle function and physical function in older women after combining resistance training and dietary modifications [12, 28]. This lack of change in strength in the current study could be attributed to the population cohort. It has been found that obese women have higher muscle strength measures when compared to lean women, which could have attributed to this lack of change [41].

Additionally the EXD group had a tendency to improve flexibility, as the participants in the EXD group improved in flexibility measures by 24% after baseline. This improvement was also demonstrated in previous studies combining Tai Chi and diet modification [15, 16]. The findings of the current study in flexibility are similar in comparison to the findings from a previous study by Katkowski et al. [15]. That 16-week study utilized Tai Chi combined with the DASH diet in obese older women, which resulted in significant within-group changes in flexibility (5.29 ± 2.16 cm; *P* = 0.022). A possible mechanism that explains these results is that Tai Chi involves the rotation of the head, trunk, and extremities while maintaining Tai Chi form, which can, therefore, improve flexibility in the arm, trunk, and hip [42].

Contrary to previous research, this study did not find significant changes in lean mass or body fat percentage between the two groups. Additionally, the current study found that anthropometric variables did not change in the EXD or CON group except for a slight, yet unexpected increase in waist to hip ratio in the EXD group. This finding is in contrast to the current literature, which has typically seen decreases in anthropometric measures after similar interventions [43, 44]. This lack of change could have occurred because the primary goal of the dietary intervention was to improve dietary quality instead of weight loss, as well as the relatively short duration of the intervention.

This study adds to the current literature on the effects of combined exercise and dietary interventions. This is also the first study to combine RT, Tai Chi, and dietary changes in obese older minority women. The findings from the current study confirm those findings in previous studies in obese older women regarding the TUG test and flexibility measures [15, 29]. However, the studies done in obese older women [15, 29] did not incorporate minority women as a major part of their study population. The current study helps fill the gap in the current literature by including this minority population and by combining multiple intervention strategies into one program.

There are several strengths of this study. First, a primary strength was the inclusion of minority women (84% non-White women). Second, the current study also included a waitlist control group that was used as a comparison and to strengthen the study design. Third, this study utilized well-validated measures of physical function in older adults.

Despite the strengths listed above, there are some limitations to this study that need to be addressed. One limitation was that the final analytical sample was only 26 total participants. However, previous studies incorporating Tai Chi that found significant results in the TUG test (1.38 seconds: *P* < 0.001) over a 12-week period had as few as 14 women in the intervention group [18]. Second, the study groups were not randomized, which could have resulted in some bias with regard to the interpretation of the results. However, there were few baseline differences between the two groups and this was primarily a translational study with the main goal of improving health of the participants. Finally, whenever a combination of intervention strategies occurs, the results cannot be attributed to a single intervention strategy. However, previous studies [15, 25, 28] have shown that each of the interventions has been shown to improve obesity-related health and function outcomes.

In conclusion, the combination of Tai Chi, RT, and diet resulted in improved TUG times and improved measures of flexibility over 12 weeks in older obese women living in an urban setting, but not in measures of muscle strength or body composition. This translational research study was able to positively influence flexibility and function of the participants and the equipment used in the study was donated to the Senior Center for continuation of the program. This study resulted in improved mobility in obese older women, and the intervention program was performed at a low cost and can easily be adopted by a senior center. Future studies should examine this combined intervention strategy with a larger sample size in this population in order to confirm these findings and to better evaluate the combined effects on muscle strength.

## Figures and Tables

**Table 1:** Baseline characteristics of the experimental group (EXD) and of the control group (CON).

Characteristic	EXD group (*n* = 17)	CON group (*n* = 9)	*P *value
Age (years)^1^	65.2 (8.1)	65.6 (8.6)	0.912
Education^2^			0.132
High school, GED, or less^∗3^	10	5	
Associates/some college^∗3^	6	1	
Bachelors or higher^∗3^	1	3	
Weight (kg)^1^	97.9 (16.1)	94.5 (12.0)	0.578
Height (cm)^1^	158.3 (6.6)	159.8 (6.0)	0.593
BMI (kg/m^2^)^1^	38.8 (5.1)	36.6 (3.4)	0.241
Waist circumference (cm)^1^	115.0 (8.9)	106.8 (9.8)	0.042
Hip circumference (cm)^1^	124.3 (11.1)	120.1 (6.2)	0.305
Waist to hip ratio^1^	0.93 (0.05)	0.89 (0.07)	0.167
Body fat (%)^1^	49.8 (3.3)	49.6 (2.9)	0.888
Race/ethnicity^1^			
Non-White^∗1^	13 (76)	9 (100)	0.263
White^∗1^	4 (24)	0 (0)	
Physical activity (kcal/week)^3,1^	7965.5 (5696.5)	3100.4 (3657.5)	0.038
Diet quality score^4,1^	72.6 (21.4)	76.4 (27.6)	0.716
At risk (<60)^*^	6 (40)	4 (44)	
Possible risk (60–75)^*^	8 (47)	4 (44)	
Not at risk (>75)^*^	2 (13)	1 (12)	

There were two baseline differences found in the physical activity kcal/week and in the waist circumference measures.

BMI: body mass index; %: percent.

All data are expressed as means with (standard deviations).

^1^Data analyzed using Student's *t*-test, ^2^data analyzed using Fisher's exact test, ^3^measured by Yale Physical Activity Survey, and ^4^measured by Dietary Screening Tool.

^*^Data are expressed as “*n*” and (percentage).

**Table 2:** Baseline data and postintervention changes in physical and muscle function in the EXD and CON groups.

Variable	EXD group (*n* = 17)	CON group (*n* = 9)	*P* value between-group
SPPB score (0–12)			
Baseline	8.4 (2.5)	8.9 (2.9)	0.628
Postintervention	9.1 (2.9)	9.0 (2.6)	
Change	0.8 (2.4)	0.5 (1.9)	0.810
4 m gait speed time (s)			
Baseline	5.41 (1.47)	5.17 (1.20)	0.681
Postintervention	5.35 (1.67)	5.17 (1.26)	
Change	−0.06 (1.00)	−0.16 (0.37)	0.781
Five-chair stand (s)			
Baseline	10.75 (5.85)	14.24 (3.87)	0.122
Postintervention	11.08 (6.35)	14.63 (5.18)	
Change	0.33 (6.24)	−0.01 (2.98)	0.624
Flexibility score (cm)			
Baseline	9.6 (10.4)	3.8 (3.9)	0.124
Postintervention	6.4 (11.1)	2.0 (6.5)	
Change	−2.3 (5.4)	−1.7 (7.0)	0.930
Knee ext. torque (kg-m)			
Baseline	6.74 (2.2)	6.65 (1.10)	0.907
Postintervention	7.67 (5.68)	6.10 (2.21)	
Change	0.92 (5.51)	−0.54 (2.54)	0.477
Grip strength (kg)			
Baseline	19.38 (6.90)	18.93 (6.06)	0.871
Postintervention	20.12 (6.57)	19.13 (5.14)	
Change	0.72 (4.09)	0.95 (3.31)	0.969

The EXD group improved flexibility by 2.3 (5.4) cm with the 12-week intervention (*P* = 0.07); however, no significant changes occurred with the 12-week intervention.

Ext.: Extension.

All data were analyzed using analysis of covariance adjusted for baseline values.

All data are expressed as least squared means (standard error).

**Table 3:** Anthropometric changes with the 12-week intervention between the EXD and CON groups.

Variable	EXD (*n* = 17)	CON (*n* = 9)	Between-group: *P* value
Weight (kg)	0.08 ± 0.69	0.34 ± 0.95	0.829
BMI (kg/m^2^)	1.10 ± 0.79	−0.34 ± 1.10	0.305
% Body fat	−0.30 ± 0.54	0.26 ± 0.74	0.549
Waist circumference (cm)	0.77 ± 1.56	3.19 ± 2.20	0.397
Waist to hip ratio	0.03 ± 0.01^*^	0.04 ± 0.01	0.542

The EXD group saw an increase in waist to hip ratio measures after the 12 weeks, and no other significant changes occurred in body composition.

^*^Significant changes within groups (*P* < 0.05). Ext: extension; %: percentage.

All data were analyzed using analysis of covariance adjusted for baseline values.

All data are expressed as least squared means (standard error).
